# Case report: Type 2 diabetes mellitus with plantar malignant melanoma: Report of two cases

**DOI:** 10.3389/fonc.2023.1089578

**Published:** 2023-03-03

**Authors:** Bi-Ling Huang, Min Tan, Ming-Liu Li, Yuan-Yuan Teng, Min Zhou, Min Wang

**Affiliations:** ^1^ Department of Endocrinology, Xiangya Hospital, Central South University, Changsha, Hunan, China; ^2^ National Clinical Research Center for Geriatric Disorders, Xiangya Hospital, Central South University, Changsha, Hunan, China

**Keywords:** type 2 diabetes, diabetic foot ulcer, malignant melanoma, case report, plantar malignant melanoma

## Abstract

Malignant melanoma is a highly malignant tumor that originates from melanocytes. It has a poor prognosis and rarely occurs on the foot. Diabetic foot ulcer is one of the most serious chronic complications of diabetes. This paper reports two cases of type 2 diabetes patients with malignant melanoma on the foot. Clinicians should improve their understanding of patients with diabetes with acral malignant melanoma. When diabetic foot ulcers occur repeatedly and continue not to heal, the clinical features of the cutaneous lesions are similar to malignant melanoma, and a pathological biopsy of the lesions should be performed promptly to obtain a clear diagnosis, avoid a missed diagnosis and improve the survival rate.

## 1 Introduction

Diabetic foot is one of the most serious chronic complications of diabetes. In severe cases, foot ulcers and gangrene, two of the main causes of diabetic nontraumatic amputation, can occur (1). High-risk factors for diabetic foot ulcers include poor blood glucose control, peripheral neuropathy, peripheral vascular disease and infection (2). Malignant melanoma is a rare, extremely malignant tumor that accounts for 5% of all skin malignancies but more than 80% of all skin cancer–related deaths (3). Some of these tumors can present as colorless ulcers (amelanotic melanoma). If such atypical melanoma occurs on the acral skin of patients with diabetes, it is easily confused with diabetic foot ulcers, which poses a challenge for the clinical diagnosis and treatment. Thus, we reviewed the related literature and clinical features of two elderly women with malignant melanoma of the foot as described below.

## 2 Case presentation

### 2.1 Case 1

**Figure 1:**
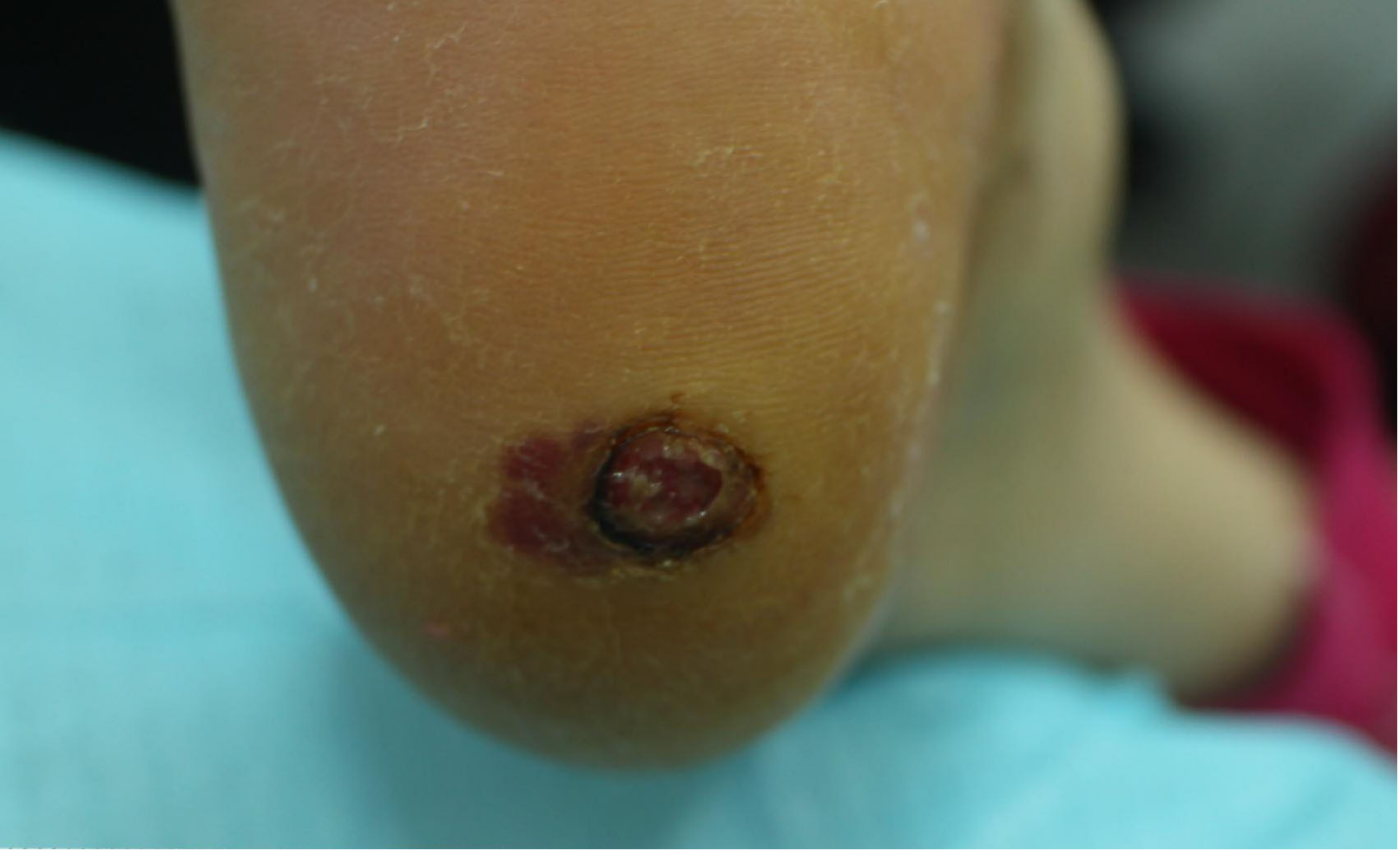
Patients’ foot wound: female, 70 years old, with right foot ulcer.

A 70-year-old woman was referred to diabetes clinic with a nonhealing ulcer of 6 months over her right heel. Medical history was positive for diabetes, hypertension and cerebral infarction. Patient denies family history of diabetes or cancer and psychosocial history. She confirmed no discomfort at the wound site. Physical examination revealed an ulcer (12 × 9 mm). On palpation, there was no warmth or tenderness in the area of the foot ulcer, and she had normal pedal pulses. Lower extremity vascular color Doppler showed the formation of multiple plaques in the lower extremity artery. After standard treatment including blood sugar control, blood pressure control, debridement and antibiotic therapy, there was no progress noted, and a black line in the shape of a half-moon appeared (**Figure 1**). Pathological biopsy showed malignant melanoma. Immunohistochemistry indicated CK5/6(-), Melan-A (+), HMB45(+), Ki67(10%+) and P63(−). The wound’s Breslow depth was 2.00mm with a Clark level 4. After discussion with the patient, she agreed to our suggestion to referred to the surgery department, and she was performed wide local excision and sentinel lymph node biopsy. Results showed that the margin was negative and there was no lymph node metastasis. Supportive care after surgery resulted in significant improvement. The patient followed up regularly every 6 months with the lesion healed and no disease recurrence until now.

### 2.2 Case 2

**Figure 2:**
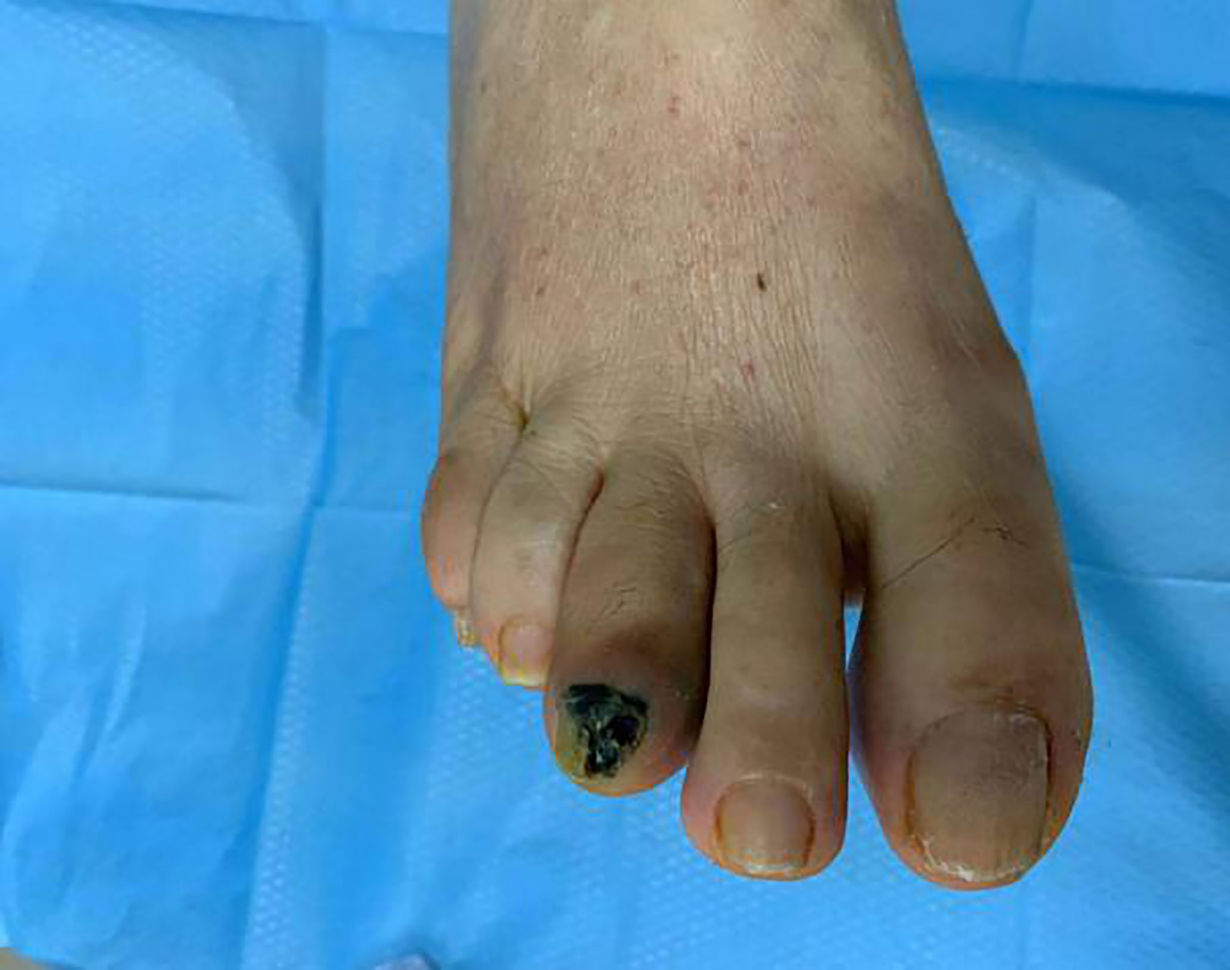
Patients’ foot wound. female,66years old, with blackening at the end of the third toe of the right foot.

A 66-year-old woman was referred to our department with non-healing foot wound over the third toe of her right foot for 2 months. Her medical history includes diabetes, chronic kidney disease and hypertension. The patient denies any family history of diabetes or cancer, as well as any psychosocial history. She was treated with local wound care, antibiotics and diabetes medication for 2 months from the local health services. Without satisfactory results, the wound remained unhealed and become black (**Figure 2**). Physical examination revealed a dark wound under the nail over the third toe of her right foot, measured 10 × 8 mm. A positive oblique radiograph of the right foot showed a bone defect of the third distal phalanx with swelling of the surrounding soft tissue. Pathological report confirmed malignant melanoma. Immunohistochemistry results were S-100(+), Melan-A (+), HMB45(+), Ki67(20%+), CD31(−) and D-20(−). The wound’s Breslow depth was unknown, and the Clark’ level was level 5. Color ultrasound demonstrated lymph node metastasis. Following a discussion with the patient, she agreed to perform radical resection of the tumor and lymph node dissection. The patient followed up regularly every 3 months after discharge, and no recurrent lesion were found temporarily.

## 3 Discussion

Diabetic foot ulcer is a serious chronic complication of diabetes and is considered a disabling ulcer that poses a great threat to the life. The International Diabetic Foot Working Group defines diabetic foot as a patient with an initial diagnosis of diabetes or a history of diabetes who has foot infections, ulcers or tissue damage (4). The main factors leading to poor prognosis are old age, high HbA1c, long course of diabetes, malnutrition, infection and so on. Additionally, when foot malignant melanoma is misdiagnosed as diabetic foot, the course of the ulcer will also be prolonged.

Melanoma is a malignant tumor caused by an uncontrolled proliferation of melanocytes. The incidence rate is approximately 2.8 to 3.1/10 million (5). Skin melanoma accounts for more than 90% of melanomas (6). According to statistics, skin melanoma accounts for 5% of all skin malignancies but accounts for more than 80% of all skin cancer–related deaths (3). Therefore, early diagnosis and treatment are of great importance for improving the prognosis of patients with malignant melanoma.

Acral lentiginous melanoma (ALM) is a rare subtype of cutaneous melanoma that occurs mainly in the palm, sole and nail bed. In people with light skin, ALM is the rarest subtype, accounting for only approximately 4%–10% of all cutaneous melanomas, whereas in people with deep skin, ALM is the most common subtype (7). ALM does not show typical ABCD signs of malignant melanoma (asymmetry, boundary, color and diameter) (8). Moreover, early-stage ALM is asymptomatic, many patients, especially the elderly, find it difficult to detect changes on the foot skin. Thus, ALM is often misdiagnosed, which delays its diagnosis and treatment.

By 2020, more than 10 cases of diabetes with plantar melanoma have been reported in English literature. Most tumors occur on the plantar, followed by the toe. Of these cases, the clinical differential diagnosis of acral malignant melanoma vary and include ulcers, warts, hematoma, nevus, granuloma and so on. Thus, the tumor is also known as the ‘great makeup artist’. Interestingly, most patients had a history of diabetes for more than 10 to 20 years. Additionally, studies have shown that chronic hyperinsulinemia increases the risk of cancer. Exogenous insulin is required in patients with type 2 diabetes and the dose usually exceeds normal insulin levels, which is also associated with an increased risk of cancer (9). Both patients in this study had a history of exogenous insulin use, and further study is needed to determine whether this is related to tumorigenesis.

The diagnosis of melanoma should rely on biopsy and immunohistochemistry. There is currently no consensus on when the pathological biopsy should be performed for intractable foot lesions. However, we recommend the following:(1) For patients with a diagnosis of diabetic foot, who have achieved blood glucose control and in whom factors causing refractory foot disease have been excluded, early pathological biopsy should be considered when the wound still does not heal or even becomes aggravated. (2) When diabetic foot lesions occur without obvious risk factors of diabetic foot disease, such as poor blood glucose control, peripheral vascular neuropathy, and trauma history, a biopsy should preferably be performed. (3) When atypical lesions, such as pigmentation and granulation tissue, are present, biopsy should be performed as early as possible for a definite diagnosis, regardless of the presence of risk factors for intractable lesions.

## 4 Conclusion

In short, acral malignant melanoma is prone to missed diagnosis because of its lack of specific clinical manifestations, and it may result in misdiagnosis in patients with diabetes. Therefore, when diabetic foot lesions with recurrent, poor healing, atypical ulcers and pigmentation were present, clinicians should perform a pathological biopsy as soon as possible to improve the survival rate of patients.

## Data availability statement

The original contributions presented in the study are included in the article/Supplementary Material. Further inquiries can be directed to the corresponding author.

## Ethics statement

The study was approved by the Ethics Committee of Xiangya Hospital of Central South University (Changsha, China). The patients/participants provided their written informed consent to participate in this study.

## Author contributions

B-LH conceived of the study and drafted the manuscript. MT and M-LL participated in the histopathological evaluation. Y-YT and MZ participated in its acquisition of data and analysis. MW participated in its design and coordination and helped to draft the manuscript. All authors contributed to the article and approved the submitted version.

## References

[1] Bandyk DF . The diabetic foot: Pathophysiology, evaluation, and treatment. Semin Vasc Surg (2018) 31(2-4):43–8. doi: 10.1053/j.semvascsurg.2019.02.001 30876640

[2] Lim JZ Ng NS Thomas C . Prevention and treatment of diabetic foot ulcers. J R Soc Med (2017) 110(3):104–9. doi: 10.1177/0141076816688346 PMC5349377 28116957

[3] Papakostas D Stefanaki I Stratigos A . Genetic epidemiology of malignant melanoma susceptibility. Melanoma Manag (2015) 2(2):165–9. doi: 10.2217/mmt.15.7 PMC6094632 30190845

[4] van Netten JJ Bus SA Apelqvist J Lipsky BA Hinchliffe RJ Game F . Definitions and criteria for diabetic foot disease. Diabetes Metab Res Rev (2020) 36(Suppl 1):e3268. doi: 10.1002/dmrr.3268 31943705

[5] Siegel RL Miller KD Jemal A . Cancer statistics, 2019. CA Cancer J Clin (2019) 69(1):7–34. doi: 10.3322/caac.21551 30620402

[6] Ali Z Yousaf N Larkin J . Melanoma epidemiology, biology and prognosis. EJC Suppl (2013) 11(2):81–91. doi: 10.1016/j.ejcsup.2013.07.012 26217116 PMC4041476

[7] Piliang MP . Acral lentiginous melanoma. Clin Lab Med (2011) 31(2):281–8. doi: 10.1016/j.cll.2011.03.005 21549241

[8] Dwyer PK Mackie RM Watt DC Aitchison TC . Plantar malignant melanoma in a white Caucasian population. Br J Dermatol (1993) 128(2):115–20. doi: 10.1111/j.1365-2133.1993.tb15138.x 8457443

[9] Vigneri P Frasca F Sciacca L Pandini G Vigneri R . Diabetes and cancer. Endocr Relat Cancer (2009) 16(4):1103–23. doi: 10.1677/ERC-09-0087 19620249

